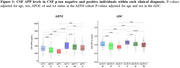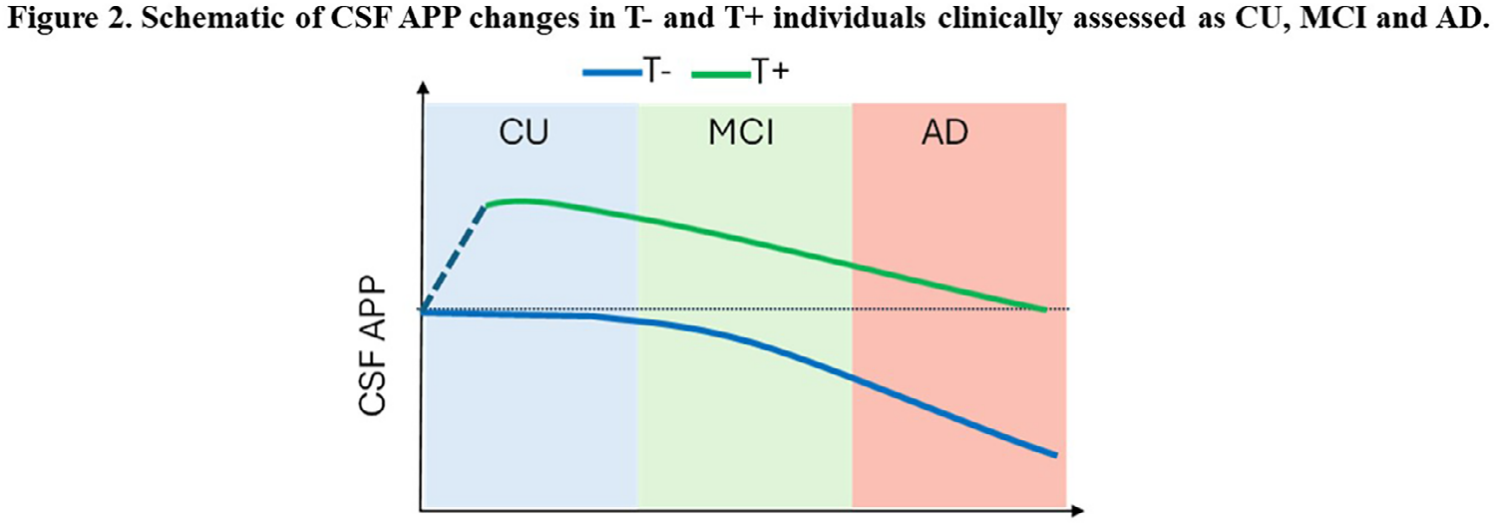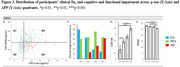# Low CSF APP refines diagnosis for *p*‐tau negative Alzheimer‐like dementia

**DOI:** 10.1002/alz70856_099406

**Published:** 2025-12-24

**Authors:** Pratishtha Chatterjee, Pawel Kalinowski, Anne M Roberts, Blaine Russell Roberts, Betty M. Tijms, Charlotte E. Teunissen, Ashley I. Bush, Scott Ayton

**Affiliations:** ^1^ The Florey Institute of Neuroscience and Mental Health, Melbourne, VIC, Australia; ^2^ Macquarie University, Sydney, NSW, Australia; ^3^ Monash University, Melbourne, VIC, Australia; ^4^ Melbourne Dementia Research Centre, Florey Institute of Neuroscience and Mental Health, The University of Melbourne, Melbourne, VIC, Australia; ^5^ Emory School of Medicine, Atlanta, GA, USA; ^6^ Emory University School of Medicine, Atlanta, GA, USA; ^7^ Amsterdam Neuroscience, Neurodegeneration, Amsterdam, North Holland, Netherlands; ^8^ Alzheimer Center Amsterdam, Department of Neurology, Amsterdam Neuroscience, Vrije Universiteit Amsterdam, Amsterdam UMC, Amsterdam, Netherlands; ^9^ Neurochemistry Laboratory, Amsterdam Neuroscience, Program Neurodegeneration, Amsterdam UMC, Vrije Universiteit Amsterdam, Amsterdam, Noord‐Holland, Netherlands; ^10^ The Florey Institute of Neuroscience and Mental Health, The University of Melbourne, Australia, Melbourne, VIC, Australia; ^11^ Florey Department of Neuroscience and Mental Health, University of Melbourne, Melbourne, VIC, Australia

## Abstract

**Background:**

The integration of biomarkers for amyloid‐β (A+) and phosphorylated tau (T+) pathology has transformed Alzheimer's disease (AD) diagnosis from being solely based on clinical assessment to a more accurate, biologically‐driven approach. However, this biomarker framework also exposes cases that deviate from the expected AD pattern, wherein 15–20% of clinically diagnosed AD cases exhibit atypical biomarker profiles (e.g. A+/T‐ dementia). This group presents a diagnostic challenge and here we investigate alternative pathophysiological mechanisms for their AD‐like symptoms.

**Methods:**

CSF amyloid precursor protein (APP) was analysed across two cohorts: ADNI (*N* = 384) and ADC (*N* = 419).

**Results:**

Concentrations of CSF APP were significantly lower in T‐ compared to T+ individuals across all clinical diagnostic groups across cohorts, regardless of A± status (Figure 1). Concentrations of APP showed a positive correlation with those of CSF *p*‐tau181 but were not associated with the Aβ42/Aβ40 ratio. APP reductions were most pronounced in T‐ cases clinically diagnosed with AD (AUC [95% CI]: 0.81 [0.70‐0.92]). Lower CSF APP levels were associated with elevated NfL, worse Mini‐Mental State Examination scores, and higher Clinical Dementia Rating‐Sum of Boxes scores. In T+ individuals, APP was initially elevated in cognitively unimpaired participants but declined with disease progression, albeit from a higher baseline (Figure 2). Stratifying participants by low/high APP and *p*‐tau revealed the highest frequency of clinical AD diagnoses in individuals with low APP and high *p*‐tau. This group exhibited the most severe cognitive and functional impairments compared to those with high APP and/or low *p*‐tau profiles (Figure 3).

**Conclusions:**

Individuals with low APP levels (APP positive) or elevated *p*‐tau181 (*p*‐tau181 positive) had the highest prevalence of AD‐like dementia, with the risk being greatest in those positive for both markers. These results reveal a unique biological profile for T‐ status in clinically diagnosed AD, who are a complex and under‐explored patient group, and highlight opportunities for enhancing diagnostic refinement. Given that APP influences neuronal plasticity, memory, synaptogenesis, and maintenance of neuronal iron homeostasis, our findings suggest that disruptions in APP production and processing may contribute to disease progression.